# Surgical management of cardiac cystic echinococcosis in a paediatric patient: a case report

**DOI:** 10.1093/icvts/ivac279

**Published:** 2022-11-24

**Authors:** Mehmet Biçer, Şima Kozan, Hüsnü Fırat Altın, Numan Ali Aydemir

**Affiliations:** Department of Pediatric Cardiovascular Surgery, Koç University Hospital, Istanbul, Turkey; School of Medicine, Koç University, Istanbul, Turkey; Department of Pediatric Cardiovascular Surgery, Dr. Siyami Ersek Thoracic and Cardiovascular Surgery Training and Research Hospital, Istanbul, Turkey; Department of Pediatric Cardiovascular Surgery, Dr. Siyami Ersek Thoracic and Cardiovascular Surgery Training and Research Hospital, Istanbul, Turkey

**Keywords:** Cystic echinococcosis, Hydatid disease, Cardiac cyst

## Abstract

Cystic echinococcosis, a zoonotic parasitic disease, is endemic to many countries worldwide. This slowly progressing disease is seen rarely in the paediatric age group. In terms of cyst localization, cardiac involvement is infrequent. We report the case of a successful surgical and medical management of a paediatric hydatid disease patient with involvement of the heart.

## CASE PRESENTATION

A 5-year-old male patient was referred to our clinic with a history of dyspnoea and exercise intolerance. In tomographic examinations, disseminated cystic lesions with the following dimensions and locations were detected: 27 mm × 40 mm located posterior to the right main pulmonary artery; 26 mm × 23 mm located in the left ventricle; 47 mm × 48 mm located posterior to the left upper lobe of the lung; in the right lung, the largest one being a 15 mm × 12 mm cyst located at superior lower lobe; 18 mm × 21 mm located at the left lobe of the liver (Video 1). Following 14 days of medical treatment of albendazole, patient was included in the elective surgical program.

Following median sternotomy, cardiac arrest was initiated using standard bypass setup at mild systemic hypothermia, cross-clamp and antegrade blood cardioplegia. Considering the risk for contamination, the heart was covered with hypertonic saline-soaked gauzes (Video 2). A solid structure was observed on the left ventricular free wall. The cystic fluid was aspirated; saline was injected into the cyst and aspirated thereafter. Then, the cyst was incised and the germinative membrane was excised. The cystic cavity was washed with 10% povidone-iodine. The cyst was plicated (capitonnage), and primary closure was performed on its outermost surface supported by Teflon felts. Cross-clamp was removed, and on the beating heart, the retro-mediastinal cyst located behind the right pulmonary artery was excised using a similar method. The patient was weaned from cardiopulmonary bypass and the chest wall was closed.

The patient was extubated on the postoperative first day. After the arrangement of his 2-year medical treatment plan with albendazole, he was discharged with surgical recovery on the postoperative seventh day. Following the completion of the medical treatment, in the 2-year postoperative follow-up of the patient, no recurrence of the cysts has been noted on the tomographic imaging (Video 3).

## DISCUSSION

The zoonotic parasitic infection cystic echinococcosis can involve all organs. Especially in children, cardiac echinococcosis is extremely rare [1, 2]. Primary echinococcosis of the heart accounts for 0.5–2% of all cases in endemic areas for cystic echinococcosis that is regions with widespread livestock production [1–5]. In like manner, our patient was from a region with livestock production.

Hydatid cysts expand slowly and remain asymptomatic until the size or space the cyst occupies has an impact on the organ affected and causes symptoms [2, 3]. In addition, symptoms of cardiac echinococcosis are non-specific, making accurate diagnosis challenging, as it was the case in our patient as well [1, 4]. The presenting symptoms will differ depending on the localization of the cyst. The most common site of involvement has shown to be the left ventricular free wall, as in our case [1, 2, 5].

Overall, surgical excision of the cysts is the preferred treatment in hydatid disease [1–3]. For operable patients, WHO guidelines recommend surgical excision of the cyst followed by a medical treatment for 2 years [2]. In inoperable patients, long-term pharmacological therapy should be provided. With regards to the medical treatment, Albendazole has been described as the first line treatment, as we have administered in this patient [1–3].

There are several complications associated with cystic echinococcosis, with the acute rupture of the cysts into the systemic circulation being the most serious among them [2, 3, 5]. A cyst rupture may generate complications of massive embolization depending on the cyst location. Also, a life-threatening anaphylactoid reaction by cause of the antigenic nature of the cystic contents is possible [3]. Thus, we believe care must be taken to avoid extensive cardiac manipulation prior to cross clamping and to secure the surgical field with saline-soaked gauzes to minimize local dissemination [1]. Additionally, we aspirated the cyst contents and washed with saline to sterilize the cavity to further help eliminate intraoperative risk of contamination [1, 3]. Hence, in this case report we highlight the significance of careful perioperative measures taken to avoid potentially fatal complications [2].

## CONCLUSION

The management of hydatid cysts needs further attention in countries endemic for cystic echinococcosis. Early diagnosis and treatment are of great importance to avoid potentially fatal consequences, especially in children. The basic treatment strategy of cardiac hydatid disease in children can be evaluated in favour of surgery. When cystic echinococcosis and its complications are considered, it will be appropriate to surgically excise all the cysts accessible during the operation. In addition, perioperative medical treatment as an adjunct to the surgical treatment is of great importance in this disease.

## ETHICS APPROVAL AND CONSENT TO PARTICIPATE

The study was approved by Koç University Committee on Human Research. Ethics approval protocol number: 2022.225.IRB1.080. Written informed consent was obtained from the patient’s guardian.

## Data Availability

The data underlying this article are available in the article and in its online supplementary material.
